# Brain-derived neurotrophic factor levels in newly diagnosed patients with bipolar disorder, their unaffected first-degree relatives and healthy controls

**DOI:** 10.1192/bjo.2021.9

**Published:** 2021-02-16

**Authors:** Nanna Aagaard Petersen, Marc Østergaard Nielsen, Klara Coello, Sharleny Stanislaus, Sigurd Melbye, Hanne Lie Kjærstad, Kimie Stefanie Ormstrup Sletved, Roger S. McIntyre, Ruth Frikke-Smith, Maj Vinberg, Lars Vedel Kessing

**Affiliations:** Copenhagen Affective Disorders Research Centre (CADIC), Psychiatric Centre Copenhagen, Rigshospitalet, Denmark; Copenhagen Affective Disorders Research Centre (CADIC), Psychiatric Centre Copenhagen, Rigshospitalet, Denmark; Copenhagen Affective Disorders Research Centre (CADIC), Psychiatric Centre Copenhagen, Rigshospitalet, Denmark; Copenhagen Affective Disorders Research Centre (CADIC), Psychiatric Centre Copenhagen, Rigshospitalet, Denmark; Copenhagen Affective Disorders Research Centre (CADIC), Psychiatric Centre Copenhagen, Rigshospitalet, Denmark; Copenhagen Affective Disorders Research Centre (CADIC), Psychiatric Centre Copenhagen, Rigshospitalet, Denmark; Copenhagen Affective Disorders Research Centre (CADIC), Psychiatric Centre Copenhagen, Rigshospitalet, Denmark; Mood Disorders Psychopharmacology Unit, University Health Network, Toronto, Ontario, Canada; Department of Clinical Biochemistry, Centre of Diagnostic Investigation, Rigshospitalet, Denmark; Copenhagen Affective Disorders Research Centre (CADIC), Psychiatric Centre Copenhagen, Rigshospitalet, Denmark; Department of Clinical Medicine, Faculty of Health and Medical Sciences, University of Copenhagen, Denmark; and Psychiatric Research Unit, Psychiatric Centre North Zealand, Hillerød, Denmark; Copenhagen Affective Disorders Research Centre (CADIC), Psychiatric Centre Copenhagen, Rigshospitalet, Denmark; and Department of Clinical Medicine, Faculty of Health and Medical Sciences, University of Copenhagen, Denmark

**Keywords:** Bipolar disorder, brain-derived neurotrophic factor, recent onset, unaffected relatives

## Abstract

**Background:**

Brain-derived neurotrophic factor (BDNF), which facilitates neuroplasticity and synaptogenesis, may be decreased in bipolar disorder, but has not been systematically investigated in people with newly diagnosed bipolar disorder and unaffected first-degree relatives.

**Aims:**

To compare BDNF levels in patients with newly diagnosed bipolar disorder, their unaffected first-degree relatives and healthy controls.

**Method:**

The study investigated plasma BDNF levels in patients (*n* = 371) with newly diagnosed bipolar disorder, their unaffected first-degree relatives (*n* = 98) and healthy controls (*n* = 200) using enzyme-linked immunosorbent assay. We further investigated associations between BDNF levels and illness-related variables and medication status.

**Results:**

BDNF levels were found to be 22.0% (95% CI 1.107–1.343) higher in patients with bipolar disorder compared with healthy controls (*P* < 0.001) and 15.6% higher in unaffected first-degree relatives compared with healthy controls (95% CI 1.007–1.327, *P* = 0.04), when adjusting for age and gender. Further, BDNF levels were positively associated with duration of illness at a trend level (*P* = 0.05), age (*P* = 0.001) and use of anti-epileptic medication (*P* = 0.05).

**Conclusions:**

These findings suggest that BDNF levels are not decreased in the early stages of bipolar disorder and in unaffected first-degree relatives contrasting with prior findings during later stages of the illness.

## Background

Bipolar disorder is often a progressive disorder with a high risk of recurrence of depressive and manic episodes^1^ and with functional disability, decreased quality of life and a reduced life expectancy of 8–12 years.^2^ It has been hypothesised that the potential pathology of bipolar disorder may be because of impairments in neuroplasticity.^3^ Brain-derived neurotrophic factor (BDNF) is a member of the neurotropic family and is widely distributed in the central nervous system. BDNF plays a central role in the survival and function of neurons in the brain^4^ and furthermore, influences the neuronal plasticity of the brain as involved in cellular models of memory and learning. Lower BDNF levels could therefore result in reduced neuronal activity, loss of normal plasticity and damage to neurons in the brain.^5^ BDNF has been demonstrated to cross the blood–brain barrier^6^ and peripheral blood levels are found to be decreased in major depression^7^ and schizophrenia.^8^

## Use of BDNF levels as a potential biomarker

As a result of these neuroplastic qualities BDNF levels have been explored in patients with bipolar disorder, and based on reviews and meta-analyses BDNF is suggested as a potential biomarker related to neuroprogression and disease activity.^9,10^ Two recent meta-analyses including 53 studies^11^ and 52 studies,^10^ found that patients with bipolar disorder tend to have lower peripheral BDNF levels during both depressive and manic episodes compared with healthy controls, and that BDNF levels may return to normal in euthymic states.

In conclusion, changes in BDNF levels seem to be involved in the pathogenesis of bipolar disorder and could reflect trait markers for the disorder, being present prior to and following onset of the illness and potentially decrease further with clinical progression of the illness.^12^ However, no large-scale study has investigated BDNF levels in people with newly diagnosed bipolar disorder and their unaffected first-degree relatives.

## Aims

The aim of our present study was to investigate plasma BDNF levels in patients newly diagnosed with bipolar disorder, and their unaffected first-degree relatives, in comparison with healthy controls. We hypothesised that patients newly diagnosed with bipolar disorder would have lower BDNF levels compared with healthy controls without a family history of bipolar disorder. Furthermore, we hypothesised that first-degree relatives of patients with bipolar disorder would express intermediary BDNF levels.

## Method

### Study design

The present cross-sectional study analyses baseline data from the ongoing, longitudinal Bipolar Illness Onset (BIO) study, which aims to identify composite biomarkers for bipolar disorder. A full research protocol has been published for the BIO cohort study.^13^ Recruitment into the BIO study started in June 2015 and ended in November 2019. The study was approved by the Committee on Health Research Ethics of the Capital Region of Denmark (protocol No. H-7-2014-007) and the Danish Data Protection Agency, Capital Region of Copenhagen (RHP-2015-023). Written informed consent was provided by all participants. The study complied with the Declaration of Helsinki principles.

### Participants

#### Patients with bipolar disorder

Patients were recruited from the Copenhagen Affective Disorder Clinic, which covers the entire greater Copenhagen catchment area (Region Hovedstaden). The Copenhagen Affective Disorder Clinic covers a catchment area of 1.6 million people, including all psychiatric centres in the region, and provides assessment and treatment for patients with newly diagnosed/first-episode bipolar disorder (i.e. for patients where the diagnosis of a single manic episode/bipolar disorder is made for the first time). All patients referred to the Copenhagen Affective Disorder Clinic with newly diagnosed bipolar disorder/a diagnosis of a single manic episode were invited to participate in the BIO study. Inclusion criteria were an ICD-10 diagnosis of bipolar disorder or a single manic episode and age 15–70 years. Patients were excluded if they had bipolar disorder secondary to brain injury.

#### Unaffected first-degree relatives

After consent was obtained from the identified patients with bipolar disorder, their siblings and offspring (age >15 years) were invited to participate in the BIO study. Inclusion criteria included being a first-degree relative of an included patient with bipolar disorder between the ages of 15–70 years. Siblings diagnosed with bipolar disorder or schizophrenia, current substance misuse, half-siblings and adopted off-springs/siblings were not included.

#### Healthy controls

Healthy controls were recruited among blood donors from the Blood Bank at Rigshospitalet Copenhagen, Denmark. The donors were contacted in the waiting room on random days. The inclusion criterion was being between the ages of 15 and 70 years, and exclusion criteria included having a personal history or first-degree family history of psychiatric disorder that required psychiatric treatment.

### Diagnostic and clinical assessment

Diagnostic assessment and an assessment of the patient's current affective state was performed by specialists in psychiatry at the Copenhagen Affective Disorder Clinic according to the ICD-10 and DSM-IV-criteria^14^ for bipolar disorder type I and bipolar disorder type II. After informed consent, PhD students in medicine or psychology subsequently confirmed the bipolar disorder diagnosis using the Schedules for Clinical Assessment in Neuropsychiatry (SCAN).^15^ Moreover, severity of depressive and manic symptoms was assessed according to the 17-item Hamilton Rating Scale for Depression Scale-17 items (HRSD-17)^16^ and the Young Mania Rating Scale (YMRS), respectively.^17^ Medication status, sleep patterns, alcohol intake, smoking habits and exercise patterns were recorded.

### Blood sample collection and analysis

Fasting blood samples were collected at the Department of Clinical Biochemistry, Rigshospitalet in a resting state between 07.30 h and 10.00 h on the same day as the clinical assessment. A total of 5 mL of blood was drawn by venepuncture into EDTA (ethylenediaminetetraacetic acid)-containing vacuum tube (Vacuette®). Afterwards, EDTA was centrifuged for 30 min at 1590 ***g*** and 4°C for 15 min. Plasma was aliquoted into Eppendorf ® tubes and kept frozen at −80°C until plasma BDNF levels were assayed. Plasma concentration of BDNF was measured in duplicate using a sandwich enzyme-linked immunosorbent assay (ELISA) kit (R&D Systems, USA, Cat. No DBNT00). The lower limit of detection for BDNF was 15.6 pg/mL. Intra-assay coefficient of variance (CV) was 4.0% and the interassay CV was 16.4%.

Seven blood samples (for five patients with bipolar disorder and two unaffected relatives) were either below the limit of detection or had too little material to be analysed and therefore could not be included. The laboratory staff was masked to the participant's diagnostic status, and the samples were randomly assigned across assays.

### Statistical analyses

To test differences between patients with bipolar disorder, their unaffected first-degree relatives and healthy controls, SPSS version 25 was used (SPSS for Windows). The level of significance was set at *P* < 0.05. Categorical clinical demographic variables were analysed with the Chi-square test, and the remaining variables by independent sample *t*-test. When assumption of normality was not met, continuous data were analysed using the non-parametric Kruskal–Wallis test and presented as median and quartiles.

Linear mixed-effect models were used in the main analyses and were performed as two separate sets (model A and model B). The analysis strategy was planned *a priori*. First, one set (model A) was performed as a between-group comparison (patients with bipolar disorder, unaffected relatives and healthy controls) in an unadjusted linear mixed-effect model, with BDNF levels as the dependent variable, and family relationship as the random effect, to account for the correlation between the related participants. Second, gender and age were added as covariates (model A-1), and third HRSD-17 (total score), YMRS (total score), alcohol consumption (>14 units per week/≤14 units per week) and smoking status (yes/no) in a fully adjusted model (model A-2), as smoking status and alcohol consumption has been suggested to influence BDNF levels when categorised as described above.^18^

In a second set (model B), we conducted multiple linear regression analyses within patients. The first adjusted model (model B-1) included age and gender and severity according to HRSD-17 and YMRS (total scores) as independent variables and BDNF levels as the dependent variable. In a second model, illness duration (years) and current psychiatric medication treatment (yes/no) with antidepressant, anti-epileptic, antipsychotic and lithium as covariates were included, in addition to affective state, as independent variables (model B-2). Afterward, the analyses were repeated but replacing the four treatment variables with the categorical variable ‘receiving psychotropic medication’ versus ‘medication-free’.

Finally, a number of *post hoc* analyses were conducted: bivariate explorative analyses to further explore the association between BDNF levels and sociodemographic and clinical variables, using correlational analyses as well as factor analysis, and *K*-means and Hierarchical cluster analysis.

For all parametric tests, BDNF levels were transformed by logarithm base to 10, and presented as back-transformed values, estimate *B*, representing the mean ratio between variables.

## Results

### Demographic and clinical characteristics

A total of 371 patients with bipolar disorder, 98 unaffected first-degree relatives and 200 healthy controls were included in the study. The majority of patients with bipolar disorder (58.6%) were in full remission defined as a score <8 on the HRSD-17 and the YMRS rating scales on the day of inclusion. Clinical and demographic variables of the participants are shown in Table 1.

**Table 1:** Demographic variables, illness characteristics, medication and plasma brain-derived neurotrophic factor (BDNF) levels in patients with bipolar disorder, their unaffected relatives and healthy controls

	Bipolar disorder group (*n* = 371)	Unaffected relative group (*n* = 98)	Healthy control group (*n* = 200)	Bipolar disorder group versus unaffected relative group, *P*	Bipolar disorder group versus healthy control group, *P*	Unaffected relative group versus healthy control group, *P*
Age, years: median (IQR)	29.1 (24.2–36.8)	26.8 (22.8–31.8)	27.4 (24.3–36.1)	0.008	0.387	0.03
Gender, female: *n* (%)	241 (65.0)	56 (57.1)	128 (64.0)	0.153	0.819	0.253
Education, total years: mean (IQR)	15 (12.5–17)	15 (13–17)	16 (14.6–17)	0.216	<0.001	0.02
HRSD-17, total score: median (IQR)	9 (5–15)	2 (0.75–4)	0 (0–2)	<0.001	<0.001	<0.001
YMRS, total score: median (IQR)	3 (0–7)	0 (0–2)	0 (0–1)	<0.001	<0.001	0.280
Weight, kg: mean (s.d.) IQR	75.9 (15.6) 43.8 to 124.6	74.4 (14.2) 51.6 to 117.2	74.3 (13.9) 49.3 to 131.4	0.387	0.246	0.991
Hight, cm: mean (s.d.) IQR	173.2 (9.0) 153 to 199	173.6 (9.3) 153 to 196	174.3 (8.8) 157 to 200	0.724	0.169	0.517
Body mass index, mean (s.d.) IQR	25.2 (4.6) 15 to 48	24.7 (4.6) 18 to 44	24.4 (3.4) 16 to 37	0.336	0.021	0.450
Current smokers, *n* (%)	160 (43.8)^a^	21 (21.9)	21 (10.5)	<0.001	<0.001	0.009
Weekly alcohol consumption, units: mean (s.d.) IQR	4.7 (6.8) 0 to 42^a^	4.7 (5.6) 0 to 22^a^	6.2 (5.5) 0 to 36	0.972	0.008	0.027
Age at onset, years: mean (s.d.) IQR	22.6 (8.6) 4 to 60	–	–	–	–	–
Illness duration, years:^a^ mean (s.d.) IQR	11.6 (8.5) 0 to 45	–	–	–	–	–
Untreated bipolar disorder, years:^b^ mean (s.d.) IQR	7.1 (8.0) −1 to 39	–	–	–	–	–
Bipolar disorder type I/type II, *n* (%)	118/253 (31.8/68.2)	–	–	–	–	–
Current affective state, *n* (%)						
Full remission	215 (58.6)	–	–	–	–	–
Mild/moderate depressive episode	90 (24.5)	–	–	–	–	–
Severe depressive episode	9 (2.4)	–	–	–	–	–
Manic episode	1 (0.3)	–	–	–	–	–
Hypomanic episode	31 (8.4)	–	–	–	–	–
Mixed episode	19 (5.2)	–	–	–	–	–
Not available	2 (0.5)	–	–	–	–	–
Current psychotropic medication^c^ (*n*, %)						
No psychotropic medication	64 (17.3)	–	–	–	–	–
Antidepressant treatment	47 (12.6)	–	–	–	–	–
Antipsychotic treatment	134 (36.0)	–	–	–	–	–
Antiepileptic treatment (*n*, %)	194 (52.2)	–	–	–	–	–
Lithium treatment	112 (30.1)	–	–	–	–	–
P-BDNF levels, pg/mL: mean (IQR)	13 035.25 (8830.49 to 18 723.59)	12 385.28 (8519.54 to 17 365.56)	11 652.75 (7277.94 to 16 605.70)	0.302	0.001	00.176

IQR, interquartile range; HRSD-17, 17-item Hamilton Ratings Scale for Depression; YMRS, Young Mania Rating Scale; P-BNPF, plasma-BNPF.

a. Illness duration defined as time from first mood episode to inclusion date.

b. Untreated bipolar disorder defined as time from first, hypomanic, manic or mixed episode to time of bipolar disorder diagnosis.

c. The percentage of current psychotropic medication exceeds 100% as several patients with bipolar disorder received more than one psychopharmaceutical and are therefore represented in more than one category.

The included 98 unaffected first-degree relatives were relatives of 83 patients with bipolar disorder, as 15 patients had two first-degree relatives included. As can be seen in Table 1, there was no statistically significant difference between patients with bipolar disorder, unaffected first-degree relatives and healthy control individuals regarding gender, height, weight and body mass index. There were minor, although statistically significant, differences in age between patients with bipolar disorder and healthy controls (29.1 *v.* 27.4 years, *P* = 0.008) and between unaffected first-degree relatives and healthy controls (26.8 *v.* 27.4 years, *P* = 0.03). Patients with bipolar disorder and their unaffected first-degree relatives had slightly fewer years of education and compared with healthy controls. Furthermore, patients with bipolar disorder and their unaffected first-degree relatives had significantly lower alcohol consumption compared with healthy controls (*P* = 0.008 and *P* = 0.027, respectively). The number of current smokers was significantly higher among patients with bipolar disorder compared with unaffected first-degree relatives (*P* < 0.001) and healthy controls (*P* < 0.001).

### Plasma BDNF levels in patients with bipolar disorder, their unaffected first-degree relatives and healthy controls

**Fig. 1:**
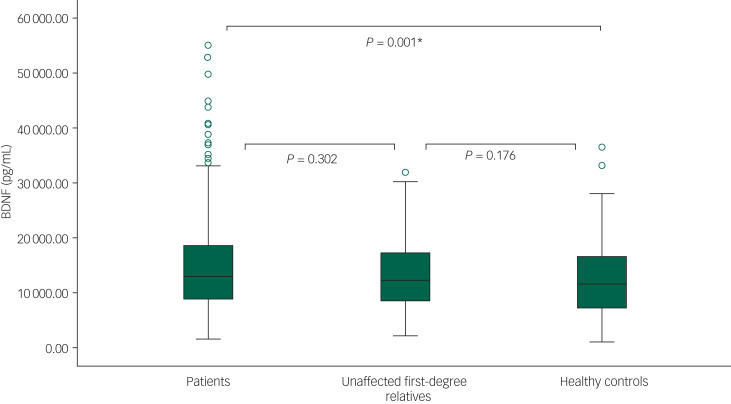
Box plots comparing brain-derived neurotrophic factor (BDNF) levels (pg/mL) in newly diagnosed patients with bipolar disorder, their unaffected first-degree-relatives and healthy controls. The lower and upper hinges represent the first and third quartiles. The upper and lower whiskers extend from the hinge to the largest and lower value, respectively. Data beyond the end of the whiskers are plotted individually. * Statistically significant at 0.05 level.

As illustrated in Fig. 1, in the unadjusted linear mixed-effect model, we found that patients with bipolar disorder had a 22.0% higher BDNF level compared with healthy controls (*B* = 1.220, 95% CI 1.104–1.343, *P* < 0.001), whereas the 8.1% higher level as compared with unaffected first-degree relatives was not statistically significant (*B* = 1.081, 95% CI 0.955–1.227, *P* = 0.214). Moreover, unaffected first-degree relatives had a 12.7% higher BDNF level than healthy controls, but this failed to reach significance (*B* = 1.127, 95% CI 0.979–1.294, *P* = 0.094).

As can be seen in Table 2, in the model 1 adjusted for group, age and gender, the BDNF levels remained statistically significantly higher in patients with bipolar disorder compared with healthy controls (*B* = 1.220, 95% CI 1.107–1.343, *P* < 0.001). Additionally, BDNF levels were 15.6% higher in unaffected first-degree relatives compared with healthy controls (*B* = 1.156, 95% CI 1.007–1.327, *P* = 0.041). There was no difference between patients with bipolar disorder and unaffected first-degree relatives (*B* = 1.054, 95% CI 0.931–1.198, *P* = 0.402). In this adjusted model, increasing age was associated with statistically significantly higher levels of BDNF (*B* = 1.008, 95% CI 1.003–1.013, *P* = 0.001). Exclusion of participants with BDNF levels higher than two s.d. above the mean (22 patients, 2 unaffected first-degree relatives and 2 healthy controls) did not change the results.

**Table 2:** Comparison of brain-derived neutrophic factor levels in newly diagnosed patients with bipolar disorder, their unaffected first-degree relatives and healthy controls

	*B*	95% CI	*P*
Model 1^a^			
Bipolar disorder versus unaffected first-degree relatives	1.054	0.931–1.198	0.402
Bipolar disorder versus healthy controls	1.220	1.107–1.343	<0.001
Unaffected first-degree relatives versus healthy controls	1.156	1.007–1.327	0.04
Age	1.008	1.003–1.013	0.001
Male versus female gender	0.998	0.911–1.094	0.967
Model 2^b^			
Bipolar disorder versus unaffected first-degree relatives	1.026	0.912–1.233	0.445
Bipolar disorder versus healthy controls	1.225	1.067–1.406	0.004
Unaffected first-degree relatives versus healthy controls	1.153	0.998–1.334	0.052
Age	1.008	1.003–1.013	0.002
Male versus female	0.997	0.906–1.098	0.951
Smoking versus not smoking	0.937	0.845–1.038	0.213
Alcohol computation (≤14 units per week versus >14 units per week)	0.927	0.790–1.088	0.354
17-item Hamilton Ratings Scale for Depression	1.003	0.995–1.012	0.432
Young Mania Rating Scale	0.998	0.986–1.009	0.683

a. Model 1: adjusted for age and gender.

b. Model 2: adjusted for age, gender, smoking, alcohol consumption and affective symptoms according to 17-item Hamilton Ratings Scale for Depression and Young Mania Rating Scale total scores.

In the fully adjusted model 2 including group, age, gender, HRSD-17 (total score), YMRS (total score), alcohol consumption and smoking status, patients with bipolar disorder had a statistically significant higher BDNF level of 22.5% compared with healthy controls (*B* = 1.225, 95% CI 1.067–1.406, *P* = 0.004, Table 2). Unaffected first-degree relatives had a statistically significant 15.3% higher BDNF level compared with healthy controls (*B* = 1.153, 95% CI 0.998–1.334, *P* = 0.05). No difference between patients with bipolar disorder and unaffected first-degree relatives were found (*B* = 1.026, 95% CI 0.912–1.233, *P* = 0.45). In the fully adjusted model, age was positively associated with BDNF levels (*B* = 1.008, 95% CI 1.003–1.0131, *P* = 0.002), but there was no association with the other covariates. These results did not change when excluding all participants with HRSD-17 ≥ 14 and YMRS ≥ 14. When participants with BDNF levels higher than two s.d.s above the mean were excluded, the significant difference between patients with bipolar disorder and healthy controls, and unaffected first-degree relatives and healthy controls, lost significance below the 0.05 level (*P* = 0.07 and *P* = 0.07, respectively).

### Associations between affective state, illness duration, medication and BDNF levels in newly diagnosed patients with bipolar disorder

As can be seen from Table 3, multiple linear regression analysis revealed no statistically significant association between BDNF levels and age, gender and symptom severity according to HRSD-17 and YMRS (total scores). However, multiple linear regression analysis with BDNF levels as the dependent variable and illness duration (years), current psychiatric medication treatment (yes/no) with antidepressants, anti-epileptics, antipsychotics and lithium as the independent variables, revealed a statistically significant association between BDNF levels and anti-epileptic medication (unstandardised *B* = 1.135, 95% CI 1.000–1.288, *P* = 0.048). Excluding patients with BDNF levels two s.d.s above the mean did not change the results. However, only 4.7% variance was explained.

**Table 3:** Model B: estimates of brain-derived neurotrophic factor levels in patients with bipolar disorder

	*B*	95% CI	*P*
Model 1, unstandardised^a^			
Age	1.007	1.000–1.012	0.074
Male versus female	1.091	0.964–1.239	0.168
17-item Hamilton Depression Rating Scale	1.002	0.984–1.009	0.771
Young Mania Rating Scale	0.995	0.984–1.007	0.455
Model 2^b^			
Age	1.002	0.993–1.009	0.794
Male versus female	1.074	0.948–1.219	0.257
Current anti-epileptic medication	1.135	1.000–1.288	0.05
Current antipsychotic medication	1.040	0.920–1.178	0.524
Current antidepressive medication	1.146	0.962–1.361	0.127
Current lithium medication	1.059	0.920–1.219	0.411
Illness duration (years)	1.005	0.995–1.014	0.239
Medication versus medication-free	0.897	0.773–1.040	0.04

a. Model 1: adjusted for age, gender and affective symptoms according to 17-item Hamilton Ratings Scale for Depression and Young Mania Rating Scale total scores.

b. Model 2: adjusted for age, gender, psychotropic medication, illness duration and medication vs non-medication.

Replacing the four treatment variables with the categorical variable ‘medication-free’ versus ‘receiving psychotropic medication’ revealed statistically significant lower BDNF levels in patients not receiving any medication (*P* = 0.04).

### *Post hoc* explorative analyses

To exclude methodological biases, exploration of variance in plasma storage time did not reveal significance (Spearman correlation, *P* = 0.1). However, there was a statistically significant positive association between longer illness duration (years) and higher BDNF levels in participants with bipolar disorder (*R* = 1.000, *P* = 0.05). Overall, independent sample *t*-tests revealed no significant difference in BDNF levels, neither between male and female gender (*P* = 0.508) nor in analysis of participant groups (bipolar disorder *P* = 0.280, unaffected relatives *P* = 0.152, healthy controls *P* = 0.08). Furthermore, when only including patients who did not receive any psychotropic medication (*n* = 64) in the mixed linear models, the difference in BDNF levels between patients with bipolar disorder and healthy controls lost significance (*P* = 0.4).

## Discussion

The BIO study is the first study to investigate BDNF in patients who are newly diagnosed with bipolar disorder or with first-episode bipolar disorder and their unaffected first-degree relatives in comparison with healthy controls. The findings did not support our first hypothesis, that newly diagnosed patients with bipolar disorder would have lower BDNF levels compared with healthy controls. In contrast, we found significantly higher BDNF levels in patients with bipolar disorder compared with healthy controls that could not be explained by alcohol consumption, smoking status or subsyndromal symptoms. Further, BDNF levels increased with illness duration. Similarly, our second hypothesis was also not confirmed as we found significantly higher BDNF levels in unaffected first-degree relatives compared with healthy controls, including when adjusting for age and gender. Overall, increasing age was associated with higher BDNF levels. Furthermore, within patients with bipolar disorder we found a significant association between receiving anti-epileptic medication and higher BDNF levels.

### Interpretation of our findings

In contrast with most prior studies investigating BDNF in patients with more progressed bipolar disorder,^10,11^ we found newly diagnosed patients to have elevated BDNF levels as compared with healthy controls. However, our finding that BDNF levels were positively associated with duration of illness (at a trend level) are in line with the previous meta-analysis from our group that included 35 studies comprising a total of 3798 participants^9^ that found higher levels of BDNF were associated with longer duration of bipolar disorder illness.

It is highly unlikely that the present findings are a result of errors in the blood sample collection or analysis process as these were undertaken according to state of the art. Fasting blood samples were collected in a resting state between 07.30 h and 10.00 h and measures of plasma concentration of BDNF were made in duplicate using a sandwich ELISA kit. To further eliminate potential methodological biases, we examined the association between BDNF levels and storage time at −80°C and found no significant association. To our knowledge, no previous studies have explored the effect of sample time after centrifugation. However, a Danish study found plasma BDNF levels to be influenced by centrifugation strategy, which was highly standardised in our analyses.^19^

BDNF levels have not previously been investigated in newly diagnosed patients with bipolar disorder. A possible explanation for our findings could be that the increased BDNF levels that we found are a result of the rapid diagnosis and subsequent medical and psychological treatment in our study. The present patients had a median age at illness onset of 20 years, and a 4-year median delay in bipolar diagnosis. The majority of the patients were in remission and were not receiving psychotropic medication at the time of inclusion and compared with the healthy control group their consumption of weekly alcohol was significantly lower (*P* < 0.001). These findings could be interpreted as a successful influence of early treatment at a highly specialised affective disorder out-patient clinic, which has a focus on psychiatric medication and group-based psychoeducation including information about the importance of abstaining from alcohol. A previous study from our group showed that patients randomised to the specialised Copenhagen Affective Disorder Clinic were treated with mood stabilisers such as lithium more often, and were substantially less rehospitalised compared with patients randomised to generalised standard treatment.^20^

The above explanation of early diagnosis and intervention resulting in increasing BDNF levels is further supported by a number of other observations. First, BDNF levels increased with illness duration in our study in accordance with similar findings in a prior meta-analysis by our group.^9^ Second, age was positively associated with BDNF levels. This finding is nevertheless in contrast to findings in prior meta-analyses.^21,22^ It should be noted that prior studies included older samples, for example, 11 of the studies included in the meta-regression by Fernandes et al^21^ had a mean age ranging from 34.0 to 48.6 years as compared with a mean age 31.4 years (median: 29.1 (quartiles: 24.2–36.8, see Table 1) in the present study.

Third, we found a significant association between higher BDNF levels and anti-epileptic medication. This is in agreement with preclinical trials showing elevated expression of BDNF in hippocampus after lamotrigine injection,^23^ and enhanced BDNF levels with valproate treatment.^24^ This may suggest that early intervention with psychotropic medication in patients with bipolar disorder may prevent early cell death, and thereby enhance neuroplasticity. This possibility is further supported by our finding of no significant difference between patients not receiving any psychotropic medication and healthy controls.

Fourth, we found significantly higher BDNF levels in unaffected first-degree relatives compared with healthy controls. This may function as a compensatory or protective effect of elevated BDNF levels in the premorbid phase in individuals at high risk of bipolar disorder in line with findings in previous studies from our group.^25,26^ Only a few studies have included the unaffected relatives of patients with bipolar disorder. Two studies suggest that unaffected relatives express higher BDNF levels compared with healthy controls without a family history of the disease.^27,28^ Nevertheless, a study examining healthy twins at high and low risk for unipolar and bipolar disorder found no association between BDNF levels and familiar risk, and BDNF levels were not associated with illness onset.^29^

When all participants were split according to gender, the significant difference in BDNF levels between patients with bipolar disorder and healthy controls was only statistically significant within women (*P* = 0.012). This may be because of differences in statistical power (men *n* = 244, women *n* = 425). However, another study from our group^26^ found women to have significantly higher BDNF levels in those individuals with a family risk of affective disorder in accordance with findings in a prior smaller study^30^ but in contrast to others.^22^ We found no significant difference between men and women in any of the three included populations, but, BDNF levels have been found to vary across time in the menstrual cycle.^30^ However, we did not add menstrual cycle as a covariate in the present study.

Notably, most patients in our study were in remission or experienced mild symptoms only reflecting that they were generally well treated. Meta-analyses have consistently found decreased BDNF levels during manic and depressive episodes and normal levels during remission^9–11^ and further that BDNF levels may increase following successful treatment of a manic episode.^10^ In addition, at least two studies that include patients with bipolar disorder who were intensively treated with mood stabilisers have found increased BDNF levels during euthymia.^31,32^ These data may further support the possibility that early medical (and psychological) intervention may add even further to increase BDNF levels as suggested by the present findings.

### Strengths and limitations

The BIO study has a number of substantial advantages. First, the blood sampling and analysis procedures were carried out in a highly standardised way, with all samples taken in a fasting state in the morning at a 3.5 h interval. Second, all samples were stored in the same manner at −80°C until plasma BDNF levels were assayed. Third, all laboratory staff was masked to participant status. Fourth, all patients with newly diagnosed bipolar disorder in the BIO study were first diagnosed clinically by a psychiatrist and then the diagnoses were verified for all participants with a SCAN interview conducted by trained PhD students in medicine or psychology. Fifth, all patients reported their current use of psychiatric medication, which was validated by trained PhD students enabling the analysis of the association between current medication status and BDNF levels. Finally, with a median age of illness onset of 20 years and a median delay in bipolar diagnosis of 4 years, the study population is representative of newly diagnosed patients with bipolar disorder. This makes it possible to investigate variables that are unaffected by long-term illness.

There are some limitations to this study. First, the healthy control group could be viewed as a particularly healthy group as they all are recruited through the blood bank, and they had higher education levels compared with patients and the unaffected first-degree relatives. Nevertheless, the blood donors included in this study were recruited from the same catchment area as patients with bipolar disorder, they did not differ in gender composition and differed only slightly in age and educational level from patients, and they were not granted economic compensation for participating. Alternative methods for recruiting control groups include using advertisements or the Danish Civil Registration System. However, both of these methods have relatively low participation response rates and a high risk of selection bias. Taken together, we believe that our control group represents the most reasonable and assessable control group for this study.

Second, although in the fully adjusted model, in addition to subsyndromal symptoms, we included variables such as smoking status and alcohol consumption that may influence BDNF levels,^18^ we cannot exclude effects of unknown or residual confounding.

Third, the number of unaffected first-degree relatives was limited compared with the patient sample, and with a median age of 27 (quartiles: 23–32) years, some may be beyond the risk of onset of bipolar disorder as the risk periods may start during adolescense. Nevertheless, the inclusion of relatives with prior and current depressive episodes increases the heterogeneity of the sample and hence the generalisability of the study. Third, the majority of the patients with bipolar disorder in this study received psychotropic medication during study participation. Consequently, we cannot entirely conclude whether the higher BDNF levels in patients compared with healthy controls is to some extent influenced by medication and not only by the disorder. Only a limited number of patients were not receiving any medication (17.3%), which may reduce the statistical power of the analyses of non-medicated patients compared with the other groups. Finally, we adjusted for a number of potential relevant confounders.

### Implications

In conclusion, we found increased BDNF levels in newly diagnosed patients with bipolar disorder and their unaffected first-degree relatives compared with healthy controls. Moreover, we found a positive association between BDNF levels, duration of illness, age and use of anti-epileptic medication. Therefore, our findings did not support our hypotheses that patients who have been newly diagnosed with bipolar disorder would have lower BDNF levels compared with healthy controls and that first-degree relatives of patients with bipolar disorder would express intermediary BDNF levels. The results contrast with prior findings of decreased BDNF levels in patients with advanced or progressed bipolar disorder and add to a more complex understanding of the possible influence of neurotrophins on the course of bipolar disorder. Findings from the study may suggest that early diagnosis and intervention may increase BDNF levels and prevent impairment of neuroplasticity.

## Data Availability

Data are available by contacting the corresponding author.
